# The Quindío stroke network: impact of an integrated care model on mortality and functional outcomes in a middle-income country

**DOI:** 10.3389/fstro.2026.1916617

**Published:** 2026-09-21

**Authors:** Laura Sofía García-Espinosa, Ángel Basilio Corredor-Quintero, Ricardo Andrés Lineros-Franco, Adriana Hernández-Cardona, Ivett Natalia Loaiza-Giraldo, Felipe Torres-Gómez, Jorge Luis Igirio-Gamero

**Affiliations:** 1 Department of Neurology, Clínica Central del Quindío, Armenia, Quindío, Colombia; 2 Essential Stroke Center, Clínica Central del Quindío, Armenia, Quindío, Colombia; 3 Internal Medicine, Cali, Valle del Cauca, Colombia

**Keywords:** cerebrovascular disease, Colombia, functional outcomes, middle-income country, mortality, reperfusion, stroke, stroke network

## Abstract

**Introduction:**

Stroke is a major cause of death and disability worldwide, with low- and middle-income countries bearing the highest burden of this condition. To address regional shortcomings in access to reperfusion therapies-particularly thrombolysis-the Red ACV-Quindío was established as the first integrated network for stroke management in central Colombia.

**Methodology:**

This pre-post quasi-experimental study evaluated the Red ACV-Quindío strategy. The study estimated functional outcomes and in-hospital mortality in patients diagnosed with stroke before and after the implementation of the network.

**Results:**

The study included a total of 841 cases. Although year-by-year variations in patient volume were detected, linear tests confirmed a homogeneous population across the study period. Baseline clinical characteristics remained stable throughout: most patients presented with minor stroke (47.1%) and low functional dependency at admission (89.0%). At discharge, overall functional independence reached 55.0% (95% CI: 51.6–58.4, *p* = 0.23) and was associated with favorable safety outcomes. Thrombolyzed patients demonstrated a significantly reduced risk of fatal outcomes (OR = 0.61; 95% CI: 0.38–0.96; *p* = 0.037). Complications were rare, with hemorrhagic transformation occurring in 4.5% of cases and malignant edema in 3.9%. In-hospital mortality showed minor fluctuations but remained under 10% overall (9.9%, *p* = 0.39).

**Discussion:**

These findings suggest that regionally integrated stroke care networks represent an effective public health intervention to mitigate stroke-associated disability and mortality in resource-limited settings.

## Introduction

Stroke is the second leading cause of death and the primary cause of acquired disability worldwide (GBD 2016 Stroke Collaborators, 2019). In Latin America, and particularly in low- and middle-income countries such as Colombia, a strong positive linear trend has been observed in the crude annual incidence rate of stroke over recent years (Kim et al., 2020). Besides the broad incidence, the general burden due to stroke is severe. According to the Colombian National Institute of Health, 16,946 deaths were attributed to cerebrovascular diseases in 2023. Of them, adults over 65 years old and the female sex were identified as most affected groups nationwide (Clínica del Occidente, 2026).

Just like mortality, post-stroke disability also constitutes a critical public health concern. The American Heart Association (AHA) estimates that between 15% and 30% of stroke survivors experience severe disability (Go et al., 2014). Particularly to Colombia, a study conducted in the Caribbean region found that 45% of patients with acute ischemic stroke presented with moderate and 22.5% with severe disability after a stroke (Chalela Blanco and Padilla Padilla, 2026). The Coffee Triangle — a subregion of Center Colombia comprising the departments of Quindío, Risaralda, and Caldas — currently lacks studies assessing functional dependence following cerebral infarction; however, data from the National Administrative Department of Statistics (DANE) consistently show that Quindío and Risaralda are historically among the departments most affected by stroke (Así Vamos en Salud, 2026). Notably, the department of Quindío recorded a mortality rate of 32.19 cases per 100,000 inhabitants in 2025, placing it among the highest in the nation (Así Vamos en Salud, 2026).

In a region severely burdened by a deadly and disabling condition for which no studies exist to adequately estimate its true impact and where the lack of a specialized care network, prolonged transfer times to referral centers located exclusively in major cities and poor stroke awareness among low-complexity facilities, condemned patients to permanent neurological sequelae or to death; a clinic in the city of Armenia — capital of the Quindío — developed in 2023, a strategy known as Red ACV-Quindío, a WhatsApp group created with the aim of directing patients with suspected stroke who are in the acute period of their symptoms to a healthcare center with the availability to provide reperfusion strategies (especially thrombolysis but also thrombectomy) and neurocritical care. The group initially formed by doctors in their mandatory social service year and a neurologist, later brought together the emergency and urgent care regulatory center (CRUE), pre-hospital care actors and personnel belonging to the emergency service of hospitals in other departments such as the north of Valle del Cauca and Risaralda.

On the instant messaging platform, it was established as a routine procedure that all patients with suspected stroke should be reported to the WhatsApp^®^ group using a pre-notification template that includes data such as last-known-well, time of symptom onset, medical and pharmacological history, among others. Based on the data presented in the chat, a radio operator from the CRUE referred the patient within minutes to the most appropriate center for treatment according to their insurance provider. During the transfer, the doctor accompanying the case, updates the changes in clinical evaluation and blood pressure every 5 min (Figure 1). In the above context, the goal of this study was to evaluate the mortality and functional dependence at discharge of patients treated since the creation of the network and compare them with the data available before its operation.

**Figure 1:**
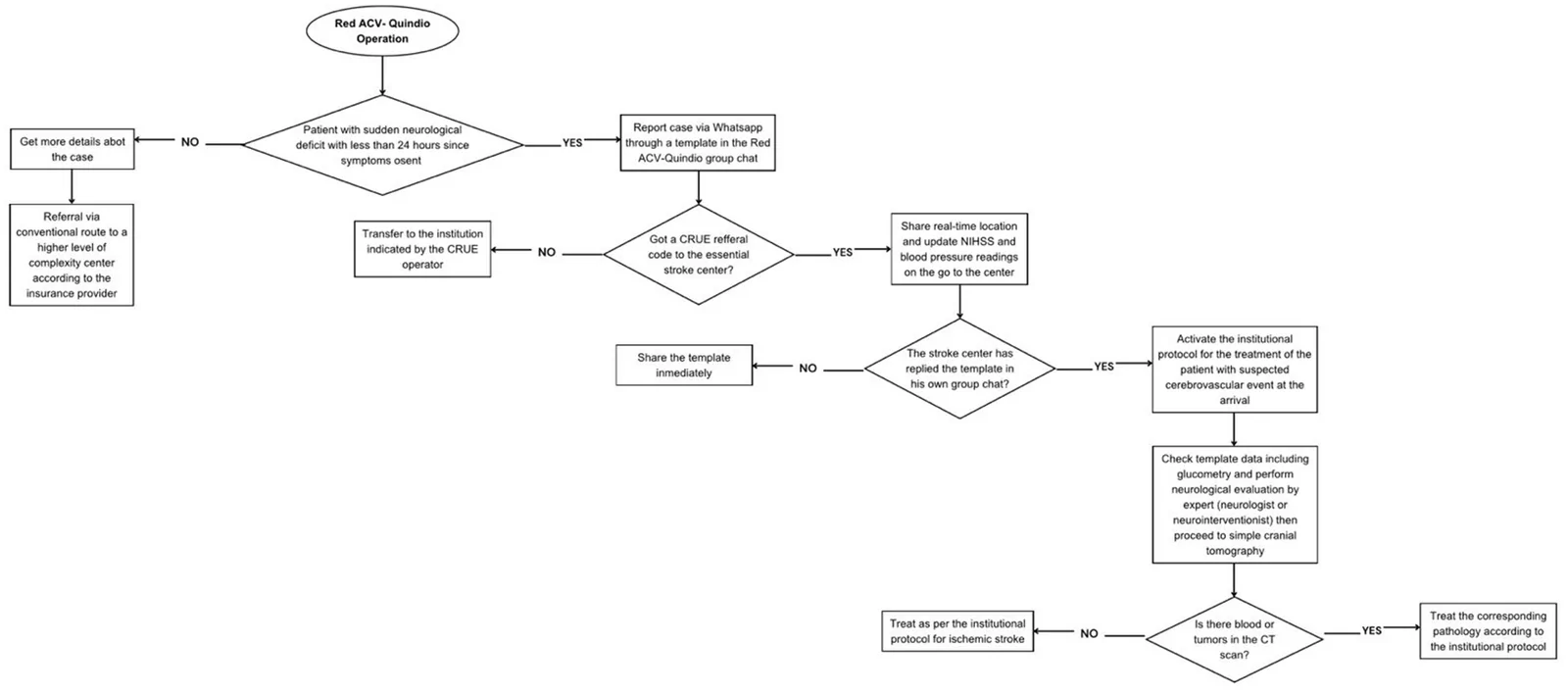
Flowchart of the general operation of Red ACV-Quindío. CT scan, computer tomography; NIHSS, national institutes of health stroke scale.

## Materials and methods

### Study design

This is a pre-post quasi experimental study that included all patients diagnosed with acute ischemic stroke who were treated at an essential stroke center certified by the World Stroke Organization (World Stroke Organization, 2026) in the city of Armenia, Quindío, between January 2022 and March 2026.

### Population

The study population included all patients with suspected stroke which time of evolution of symptoms was less than 24 hours, that had either spontaneously consult to the stroke center or been referred to the institution by the CRUE after being discussed in the Red ACV-Quindío WhatsApp^®^ group.

At the referral center, cases where their final diagnosis following clinical and imaging corresponded to hemorrhagic stroke of any subtype, transient ischemic attack, intracranial tumor or stroke mimic, were excluded. Eligibility for thrombolysis was not an inclusion criterion (Figure 2).

**Figure 2:**
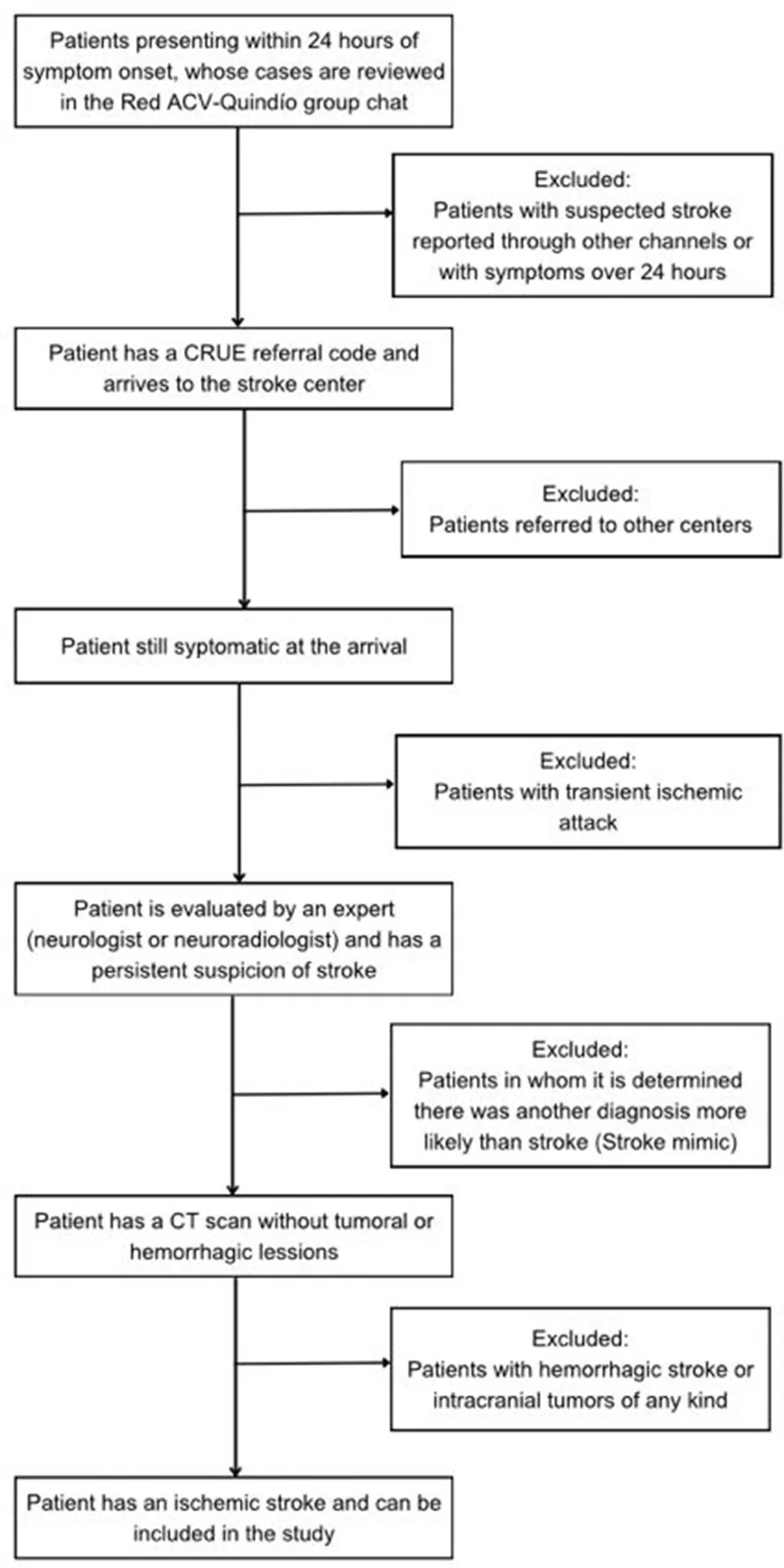
Flowchart of the patient's selection.

The decision to select patients only during the acute symptom period (less than 24 h of evolution) was based not only on the eventual opportunity for reperfusion therapies but also on recommendations from international guidelines on neurocritical care (Powers, 2019). Patients with more than 24 hours of symptoms received medical attention but were not admitted through the Red ACV-Quindío strategy and were therefore excluded from the analysis.

A sample size calculation was not performed since all cases who met the inclusion criteria were included in the study. The total number of patients in the study population were registered on the Stroke Care Quality Registry (RES-Q) platform.

### Data collection

Data were analyzed from medical records of low- and medium-complexity referring health facilities, messages in the Red ACV-Quindío WhatsApp^®^ group, as well as from the clinical records generated at the receiving institution. In the latter, patients were identified using the following ICD-10 codes: I630, I631, I632, I633, I634, I635, I636, I637, I638, and I639.

Once the relevant data were obtained, they were organized in a Microsoft Excel spreadsheet, in which each participant was assigned an alphanumeric identification code.

The messages evaluated in the WhatsApp^®^ group contain the items specified in Figure 3.

**Figure 3:**
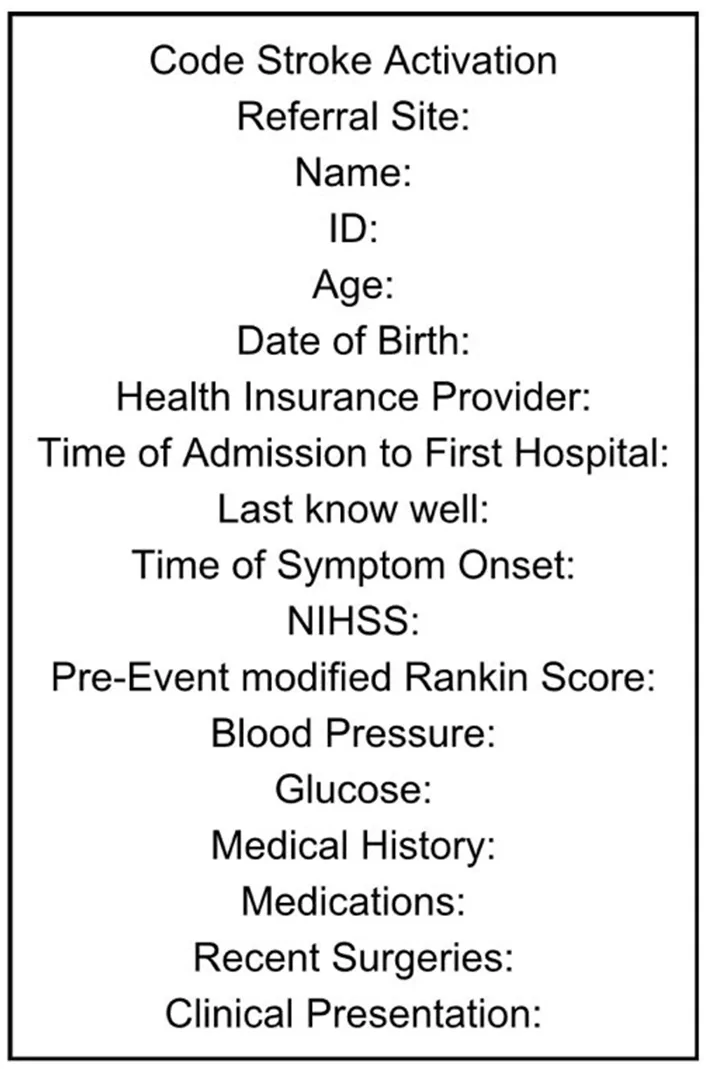
Template of Code Stroke Activation in the Red ACV-Quindío WhatsApp^®^ group. NIHSS, national institute of health stroke scale/score.

### Variables analyzed

Among sociodemographic variables, age, sex, comorbidities, and geographic origin were assessed for each case. Patients were stratified into three age groups: under 50 years, between 50 and 65 years, and over 65 years. Medical history was categorized according to classically described modifiable cardiovascular risk factors (e.g., arterial hypertension, type 2 diabetes mellitus, dyslipidemia, and smoking; 9, 10), as well as other comorbidities.

The nature of the cerebrovascular event was classified as ischemic or other, with other being the sum of patients diagnosed with hemorrhagic stroke, transient ischemic attack (TIA), and stroke mimic.

The neurological deficit was measured using the National Institutes of Health Stroke Scale (NIHSS) and was assessed at both admission and discharge for each patient by a professional trained in that matter by the World Stroke Organization (WSO) guidelines available. It was considered that patients with 0 to 4 points corresponded to a minor stroke, 5 to 15 a moderate, 16 to 20 a moderate to severe and 21 to 42 severe.

Regarding reperfusion therapies, the thrombolysis rate, the fibrinolytic agent used, the administered dose, and the door-to-needle time — the interval between patient arrival at the center and administration of the fibrinolytic bolus — were evaluated. Pre-notification was defined as the act by which the receiving center was alerted in advance of an incoming patient's arrival via WhatsApp group, and this rate was compared against the thrombolysis rate. These numbers were benchmarked against national statistics recorded for Colombia in the RES-Q platform. The eligibility for thrombolysis was taken as 4.5 h from last known well due to the unavailability of perfusion studies and others that would allow for its extension (Powers, 2019). Data on mechanical thrombectomy were not included as it will be the topic of future research.

Additionally, the rate of hemorrhagic transformation (HT) and malignant edema were measured. The radiographic definition of HT was made using the European Cooperative Acute Stroke Study (ECASS) on a CT scan taken 24 h post thrombolysis or earlier according to patient condition (Fiorelli et al., 1999). HT was considered symptomatic when it was associated with an increase of at least four points in the NIHSS scale (Fiorelli et al., 1999).

Finally, for each year of the study period, the overall in-hospital mortality rate was determined, and the modified Rankin Scale (mRS) was assessed at both admission and discharge for each patient by a professional trained in that matter by the WSO guidelines available.

### Statistical analysis

Statistical analysis was performed in R version 4.3.2 (R Core Team, Vienna, Austria) using the DescTools and exact 2 × 2 packages, with a fixed random seed for all stochastic procedures.

For categorical variables, absolute frequencies and percentages were reported with 95% confidence intervals calculated by the Wilson score method, preferred over the normal approximation because of small numerators. NIHSS scores were available as individual values for each patient and were summarized as medians with interquartile ranges computed directly from the raw scores; no interpolation of grouped categories was performed. Severity was additionally presented as ordinal frequency categories. Because the NIHSS is an ordinal scale, non-parametric methods were used throughout: distributions across years were compared with the Jonckheere-Terpstra test for trend, and within-patient change between admission and discharge was assessed with the Wilcoxon signed-rank test.

Differences across the five study periods (2022, 2023, 2024, 2025, and 2026 Q1) were assessed by the χ^2^ test of independence when all expected cell frequencies reached 5, and by the Fisher-Freeman-Halton exact test otherwise, with *p* values obtained by Monte Carlo simulation using 2 × 10^5^ replicates.

Because the study hypothesis concerned progressive change following implementation of the Red ACV-Quindío strategy rather than heterogeneity alone, trend across the study period was assessed as the primary inferential result. For dichotomous outcomes, the Cochran-Armitage test for linear trend was applied. Associations were quantified as odds ratios with Woolf 95% confidence intervals and Fisher exact *p* values, using 2022 as the reference period. Comparisons against national RES-Q data are descriptive, as the published figures are aggregate estimates without measures of dispersion.

Patients with undocumented outcome scores were excluded from the corresponding denominators in very specific cases and two sensitivity analyses were performed.

All tests were two-sided with a significance threshold of 0.05. No correction for multiple comparisons was applied, and associations reaching nominal significance in unadjusted analyses are presented as hypothesis-generating rather than as independent effects.

### Ethical considerations

This study was approved by the Bioethics Committee of the Clínica Central del Quindío through Resolution No. 02, dated May 20, 2026.

Before joining the Red ACV-Quindío WhatsApp group, all members agreed to use the platform solely for discussing patients and not to send messages of any other nature. Additionally, the identification data provided there was used only by the CRUE operator to verify medical coverage, insurance provider, and origin, and based on this information, direct cases appropriately.

## Results

Data were collected from 1,381 patients with clinical suspicion of stroke. Of these, 841 corresponded to ischemic events and were included in the analysis. The remaining 540 patients corresponded to the sum of TIA, mimic stroke, and hemorrhagic events of any type (Table 1).

**Table 1:** Patient's volume and frequency of ischemic events by year.

Year	2022	2023	2024	2025	2026 (Q1)	Conglomerate
Ischemic stroke *n* (%)	68 (64.8)	253 (63.1)	294 (59.3)	162 (58.7)	64 (62.1)	841 (60.9)
Other types of strokes *n* (%)	37 (35.2)	148 (36.9)	202 (40.7)	114 (41.3)	39 (37.9)	540 (39.1)
Total	105	401	496	276	103	1,381

### Patient volume

In 2022 — prior to the establishment of the Red ACV-Quindío strategy — a total of 68 cases of cerebral infarction were attended. By 2023, following certification as an essential stroke center, the number of patients treated increased to 401. In 2024, patient volume continued to grow, reaching a historic high of 496 events. Despite this upward trend, 2025 saw a 45% decline in total volume, attributable to factors discussed later in this report. Regarding the first quarter of 2026, 103 cases were attended during this period, with a mean of at least one case per day.

The ischemic component accounted for most presentations, with a consistently proportional fraction exceeding 50% across all study periods.

### Sociodemographic characteristics

Of the 841 included patients, 50.4% were female and 91.7% came from urban areas of the department. The median age was 72 years (IQR 63–81), with the youngest patient being 17 years old and the oldest 96. More than two-thirds of patients were older than 65 years.

Regarding comorbidities, 73.1% of patients had a known diagnosis of arterial hypertension (HTN), and 145 had a prior cerebrovascular event of any nature. The concurrent presence of diabetes mellitus (DM), HTN, and dyslipidemia was observed in 13.4% of the total population. Other clinically relevant comorbidities included hypothyroidism, chronic obstructive pulmonary disease (COPD), atrial fibrillation, and malignancy of any type (Table 2).

**Table 2:** Sociodemographic characteristics of included patients, stratified by year.

Variable	Category of variable	2022 (N: 68)	2023 (N:253)	2024 (N:294)	2025 (N:162)	2026 (Q1) (N: 64)	Conglomerate (N: 841)
Sex *n* (%)	Feminine	35 (51.5)	125 (49.4)	149 (50.7)	85 (52.5)	30 (46.9)	424 (50.4)
	Masculine	33 (48.5)	128 (50.6)	145 (49.3)	77 (47.5)	34 (53.1)	417 (49.6)
Age *n* (%)	< 50 years	2(2.9)	16 (6.3)	24 (8.2)	14 (8.6)	1 (1.6)	57 (6.7)
	50–65 years	12 (17.7)	63 (24.9)	69 (23.5)	50 (30.9)	15 (23.4)	209 (24.9)
	>65 years	54 (79.4)	174 (68.8)	201 (68.4)	98 (60.5)	48 (75.0)	575 (68.4)
Origin *n* (%)	Urban	34 (50.0)	236 (93.3)	281 (95.6)	156 (96.3)	64 (100.0)	771 (91.7)
	Rural	34 (50.0)	17 (6.7)	13 (4.4)	6 (3.7)	0 (0.0)	70 (8.3)
Risk factors *n* (%)	Hypertension	53 (77.9)	182 (71.9)	218 (74.1)	113 (69.7)	49 (76.6)	615 (73.1)
	Diabetes mellitus	12 (17.6)	57 (22.5)	73 (24.8)	51 (31.5)	22 (34.4)	215 (25.6)
	Dyslipidemia	22 (32.3)	73 (28.8)	107 (36.4)	42 (25.9)	21 (32.8)	265 (31.5)
	Active smoker (last 10 years)	8 (11.8)	7 (2.8)	1 (0.3)	6 (3.7)	0 (0.0)	22 (2.6)
	Previous stroke	13 (19.1)	42 (18.6)	48 (16.3)	32 (19.7)	10 (15.6)	145 (17.2)
	HTN + DM + Dyslipidemia	9 (13.2)9	19 (7.5)	60 (20.4)	17 (10.5)	8 (12.5)	113 (13.4)
Other comorbidities *n* (%)	Malignancy (any type)	0 (0.0)	2 (0.8)	8 (2.7)	3 (1.8)	3 (4.7)	16(1.9)
	Atrial fibrillation	1 (1.5)	2 (0.8)	4 (1.4)	5 (3.1)	2 (3.1)	14 (1.7)
	COPD	0 (0.0)	3 (1.2)	18(6.1)	9 (5.5)	3 (4.7)	33 (3.9)
	Hypothyroidism	0 (0.0)	3 (1.2)	35 (11.9)	17 (10.5)	10 (15.6)	65 (7.7)

HTN, arterial hypertension; DM, diabetes mellitus; COPD, chronic obstructive pulmonary dissease.

### Severity of stroke

Stroke severity at admission stayed essentially unchanged throughout the study period. Nearly half of the patients presented with minor stroke (NIHSS 1–4) at first assessment (47.1%) and annual proportions ranged from 42.7% in 2023 to 52.7% in 2024. Moderate strokes (NIHSS 5–15) were the next largest group (335 patients, 39.8%), while severe presentations were consistently uncommon with less than 10% of frequency in all years (6.1% scored 21–42). The estimated median NIHSS at admission was 5 (IQR 2–11) for the whole cohort, and it moved only between 4 and 6 in the individual years. The Cochran-Armitage trend test gave a p value of 0.28, which was not statistically significant (Table 3a).

**Table 3a:** Severity of stroke during the study period.

Variable	NIHSS range	2022 (N: 68)	2023 (N:253)	2024 (N:294)	2025 (N:162)	2026 (Q1) (N: 64)	Conglomerate (N: 841)	*p* trend^a^
NIHSS at admission *n* (%)	1-4	31 (45.6)	108 (42.7)	155 (52.7)	72 (44.5)	32 (50)	396 (47.1)	
	5-15	30 (44.1)	107 (42.3)	106 (36.1)	70 (43.2)	22 (34.4)	335 (39.8)	
	16-20	4 (5.9)	19 (7.5)	16 (5.4)	13 (8.0)	5 (7.8)	57 (6.8)	
	21-42	3 (4.4)	19 (7.5)	17 (5.8)	7 (4.3)	5 (7.8)	51 (6.1)	
	Median [IQR]^b^	6 [3–11]	5 [3–12]	4 [2–10]	5 [3–10]	4 [2–11]	5 [2–11]	0.28
NIHSS at discharge *n* (%)	0-4	30 (44.1)	160 (63.2)	199 (67.7)	112 (69.1)	44 (68.7)	545 (64.8)	
	5-15	16 (23.5)	44 (17.4)	50 (17)	35 (21.6)	10 (15.6)	155 (18.4)	
	16-20	1 (1.5)	9 (3.6)	5 (1.7)	1 (0.6)	1 (1.6)	17 (2.1)	
	21-42	2 (2.9)	21 (8.3)	16 (5.4)	5 (3.1)	0 (0)	44 (5.2)	
	Deceased^c^	0 (0.0)	18 (7.1)	31 (10.5)	12 (7.4)	9 (14.1)	70 (8.3)	
	NA (sedated)^d^	0 (0.0)	1 (0.4)	2 (0.7)	0 (0.0)	0 (0.0)	3 (0.4)	
	ND (no data)^e^	19 (27.9)	11 (4.3)	2 (0.7)	1 (0.6)	0 (0.0)	33 (3.9)	
	Median [IQR]^b, f^	2 [0–8]	2 [1–6]	2 [1–4]	2 [1–4]	2 [0–4]	2 [1–5]	0.093

^a^Jonckheere-Terpstra test for trend across years; severity did not vary significantly at admission or discharge.

^b^Median and interquartile range computed from individual patient scores, not from interpolation of grouped categories. Discharge values are restricted to surviving, evaluable patients.

^c^In-hospital deaths, reported as a mortality outcome in Table 8 and excluded from the discharge median.

^d^NA: NIHSS not assessable due to sedation.

^e^N.D.: no NIHSS recorded in the clinical history.

^f^Wilcoxon signed-rank test, admission vs. discharge within the same surviving patient. Exact values: 2022 p = 3.1 × 10^−3^; 2023 p = 2.9 × 10^−18^; 2024 p = 3.7 × 10^−20^; 2025 p = 2.4 × 10^−14^; 2026 Q1 p = 4.4 × 10^−6^; overall p = 1.0 × 10^−55^.

At discharge, the proportion of patients leaving the center with little or no deficit (NIHSS 0–4) went from 44.1% in 2022 to 68.7% in the first quarter of 2026, with 545 patients (64.8%) in this category overall. The 16–20 group stayed at or below 3.6% every year of the study and in the severe categories is notable that nobody was discharged with an NIHSS of 21–42 in 2026, compared with a peak of 8.3% in 2023. The median discharge NIHSS was 2 in every year. As with admission, the distribution showed no significant variation across periods (*p* = 0.093). In-hospital deaths (70 patients without a discharge score, plus 13 who died with a score recorded, totaling 83) were treated as a separate mortality outcome and excluded from the discharge severity distribution.

When interpatient analysis was made, in every period, surviving patients improved markedly between admission and discharge, with the median NIHSS falling from 4–5 to 2 (Wilcoxon *p* < 0.001 in all years except 2022, *p* = 0.003; overall *p* < 0.001) (Table 3b).

**Table 3b:** Intra-patient change (admission vs. discharge), survivors.

Scenario	2022 (N: 68)	2023 (N:253)	2024 (N:294)	2025 (N:162)	2026 (Q1) (N: 64)	Conglomerate (N: 841)
Median (admission vs. discharge)	4 → 2	5 → 2	4 → 2	5 → 2	4 → 2	4 → 2
*p* ^f^	0.003	< 0.001	< 0.001	< 0.001	< 0.001	< 0.001

^f^Wilcoxon signed-rank test, admission vs. discharge within the same surviving patient. Exact values: 2022 p = 3.1 × 10^−3^; 2023 p = 2.9 × 10^−18^; 2024 p = 3.7 × 10^−20^; 2025 p = 2.4 × 10^−14^; 2026 Q1 p = 4.4 × 10^−6^; overall p = 1.0 × 10^−55^.

A sensitivity analysis confirmed these findings and suggested the improvement is therefore not an artifact of missing data (Table 3c).

**Table 3c:** Sensitivity analysis of functional independence at discharge (NIHSS 0–4) to the handling of no data (ND) cases.

Scenario	2022 (N: 68)	2023 (N:253)	2024 (N:294)	2025 (N:162)	2026 (Q1) (N: 64)	Conglomerate (N: 841)	*p* ^a^
Complete-case (survivors evaluated) — primary % [95% Wilson confidence interval]	30/49 61.2% [47.2–73.6]	160/223 71.7% [65.5–77.3]	199/259 76.8% [71.3–81.6]	112/149 75.2% [67.7–81.4]	44/55 80.0% [67.6–88.4]	545/735 74.1% [70.9–77.2]	0.034
Scenario A: all ND = unfavorable (worst case) % [95% Wilson confidence interval]	30/68 44.1% [32.9–55.9]	160/234 68.4% [62.2–74.0]	199/261 76.2% [70.7–81.0]	112/150 74.7% [67.2–81.0]	44/55 80.0% [67.6–88.4]	545/768 71.0% [67.7–74.1]	< 0.001
Scenario B: all NA = favorable (best case) % [95% Wilson confidence interval]	49/68 72.1% [60.4–81.3]	171/234 73.1% [67.1–78.4]	201/261 77.0% [71.5–81.7]	113/150 75.3% [67.9–81.5]	44/55 80.0% [67.6–88.4]	578/768 75.3% [72.1–78.2]	0.243
ND cases, *n*	19	11	2	1	0	33	

Deaths and sedated patients are excluded from all scenarios (deaths reported as mortality, sedation as non-assessable); the sensitivity analysis varies only the handling of no-data cases. Each cell shows favorable/denominator — proportion.

^a^Cochran-Armitage test for linear trend across periods.

### Thrombolysis

The pharmacological reperfusion data showed that thrombolysis rates rose from 45.6% (95% CI 34.3–57.3) in 2022 to 64.2% (95% CI 56.6–71.2) in 2025, with a global rate of 52.1% (95% CI 48.7–55.4) across 438 of 841 ischemic events (Table 4). The test of independence (*p* = 0.005) and the Cochran-Armitage test for linear trend (*p* = 0.001) demonstrated a temporal increase rather than random fluctuation. The odds of receiving thrombolysis in 2025 were more than twice those of 2022 (OR 2.14, 95% CI 1.20–3.80), the only pairwise comparison reaching statistical significance.

**Table 4:** Thrombolysis indicators at the essential stroke center vs. national data from the RES-Q platform.

Variable	Indicator	2022 (N: 68)	2023 (N:253)	2024 (N:294)	2025 (N:162)	2026 (Q1) (N: 64)	Conglomerate (N: 841)
Thrombolysis rate	n/N	31/68	121/253	144/294	104/162	38/64	438/841
	% [95% CI]^a^	45.6 [34.3–57.3]	47.8 [41.7–54.0]	49.0 [43.3–54.7]	64.2 [56.6–71.2]	59.4 [47.1–70.5]	52.1 [48.7–55.4]
	OR vs. 2022 [95% CI]	1.00 (ref)	1.09 [0.64–1.87]	1.15 [0.67–1.95]	2.14 [1.20–3.80]	1.74 [0.87–3.48]	
	Colombia, RES-Q (%)	16	20	21	20	23	
	Difference (pp)	+29.6	+27.8	+28.0	+44.2	+36.4	
	Rate ratio	2.85	2.39	2.33	3.21	2.58	
	*p* vs. national^b^	< 0.001	< 0.001	< 0.001	< 0.001	< 0.001	
Door-to-needle time (mean, min)	Essential stroke center	20	21	28	27	38	
	Colombia (RES-Q)	57	50	50	49	47	
	Difference (min)	−37	−29	−22	−22	−9	
Dose of thrombolytic agent (mean, milligrams)^*^	Essential stroke center	61.2	61.5	59.9	61.9	61.5	
	Colombia (RES-Q)	n.d.	59.9	57.6	57.7	55.6	
	Difference (mg)	—	+1.6	+2.3	+4.2	+5.9	

χ^2^ test of independence across years (thrombolysis rate) = 15.01, *df* = 4, *p* = 0.005; Cramér's *V* = 0.134. Cochran-Armitage test for linear trend: *z* = 3.19, *p* = 0.001.

^a^Wilson score interval. Numerators reconstructed from reported percentages and annual denominators.

^b^Exact binomial test, one-sample, against the national proportion for the corresponding year.

n.d., not reported by the RES-Q platform. No inferential testing was applied to door-to-needle time or thrombolytic dose, as measures of dispersion were unavailable.

^*^Data from Alteplase use only.

When comparing annual reperfusion strategy outcomes against national data for Colombia available on the RES-Q platform, a thrombolysis rate consistently higher than the national mean was observed. The center exceeded the Colombian average by 22 to 44 percentage points in every year, corresponding to rate ratios between 2.33 and 3.21, with exact binomial tests yielding *p* values below 0.001 throughout. However, these tests treat the national figure as a fixed, error-free constant, which is not (the RES-Q value is itself an estimate derived from participating centers, with their own sampling variability and its own selection biases regarding which institutions report). Therefore, the *p* values correspond to a measure of the magnitude of the gap rather than a formal hypothesis tests.

Regarding door-to-needle time, this increased progressively throughout the study period from a mean of 20 min in 2022 to 38 min in 2026. Despite this, the center remained below the national average throughout, and all values stayed within the recommended 60-min threshold. The rise in the time of access to thrombolysis will be discussed later in the text.

Finally, the mean thrombolytic remained stable at approximately 60 mg throughout, consistently exceeding the national average by 1.6 to 5.9 mg. The most frequently used fibrinolytic agent was alteplase, administered in 97% of cases.

### Complications after stroke

As said before, two complications were evaluated: hemorrhagic transformation and malignant edema. About HT, this occurred in 38 patients (4.5%, 95% CI 3.3–6.1) and edema in 33 (3.9%, 95% CI 2.8–5.5). After inferential statistics calculations, there was no significant variation between years (*p* = 0.32 and *p* = 0.21) and no linear trend in the two categories (Table 5a).

**Table 5a:** Hemorrhagic transformation and malignant edema by year.

Variable	Presence/ ausence	2022 (N:68)	2023 (N:253)	2024 (N:294)	2025 (N:162)	2026 (Q1) (N:64)	Conglomerate (N:841)	*p* ^a^
Hemorrhagic transformation	Yes No *n* (%)	4 (5.9) 64 (94.1)	9 (3.6) 244 (96.4)	11 (3.7) 283 (96.3)	12 (7.4) 150 (92.6)	2 (3.1) 62 (96.9)	38 (4.5) 803 (95.5)	0.32
	After thrombolysis n/n	4/4	9/9	8/11	9/12	2/2	32/38 (84.2)	
	Symptomatic n/n	0/4	1/9	1/11^c^	1/12^d^	1/2^e^	4/38 (10.5)	
	95% CI^b^	2.3–14.2	1.9–6.6	2.1–6.6	4.3–12.5	0.9–10.7	3.3–6.1	
Malignant edema	Yes	2 (2.9)	16 (6.3)	9 (3.1)	5 (3.1)	1 (1.6)	33 (3.9)	0.21
	No	66 (97.1)	237 (93.7)	285 (96.9)	157 (96.9)	63 (98.4)	808 (96.1)	
	95% CI^b^	0.8–10.1	3.9–10.0	1.6–5.7	1.3–7.0	0.3–8.3	2.8–5.5	

^a^Fisher-Freeman-Halton exact test across years (Monte Carlo, 2 × 10^5^ replicates). Cochran-Armitage trend: hemorrhagic transformation *p* = 0.58; malignant edema *p* = 0.12.

^b^Wilson score interval.

^cde^NIHSS could not be calculated in 5, 4 and 1 cases respectively because of sedation.

A pooled comparison by reperfusion status was made regarding the hemorrhage finds. Among treated patients, hemorrhagic transformation occurred in 7.3% vs. 1.5% of untreated patients (OR 5.22, 95% CI 2.16–12.61). Only 4 of the 38 transformations were symptomatic which represents less than 1 % of the entire population (0.48% with 95% CI 0.19–1.22) (Table 5b). The particularities of the interpretation of these results are later discussed.

**Table 5b:** Pooled comparison by reperfusion status.

Complication	Thrombolyzed (*n* = 438)	Non-thrombolyzed (*n* = 403)	OR (95% CI)	*P*
Hemorrhagic transformation	32 (7.3)	6 (1.5)	5.22 (2.16–12.61)	< 0.001^f^

^f^Fisher exact test. Denominators derived from data reported in Table 4.

### Pre-notification

Following certification as an essential stroke center and the establishment of the Red ACV-Quindío network, a shift in the admission pathway of ischemic stroke patients into the healthcare system was observed. The prenotification, widely used in the first years of the study, declined significantly over time. After rising from 45.6% in 2022 to a peak of 57.1% in 2024, it fell to 35.8% in 2025 and 29.7% in the first quarter of 2026 (trend *p* < 0.001). Conversely, walk-in patients — defined as those arriving at the center by their own means — gained increasing relevance, accounting for more than 70% of admissions during the first quarter of 2026 (Table 6). Among the patients considered as non-pre-notified, for practical analytical purposes, in-hospital stroke cases were also classified as.

**Table 6:** Thrombolysis vs. pre-notification by year.

Variable	Presence/ ausence	2022 (N: 68)	2023 (N:253)	2024 (N:294)	2025 (N:162)	2026 (Q1) (N: 64)	Conglomerate (N: 841)	*p* ^a^
Pre-notification *n* (%)	Yes	31 (45.6)	136 (53.8)	168 (57.1)	58 (35.8)	19 (29.7)	412 (49.0)	< 0.001
	95% CI^b^	34.3–57.3	47.6–59.8	51.4–62.7	28.8–43.4	19.9–41.8	45.6–52.4	
	No	37 (54.4)	117 (46.2)	126 (42.9)	104 (64.2)	45 (70.3)	429 (51.0)	
Thrombolysis *n* (%)	PN n/n (%)	16/31 (51.6)	81/136 (59.6)	75/168 (44.6)	38/58 (65.5)	10/19 (52.6)	220/412 (53.4)	0.96
	95% CI^b^	26.3–56.5	26.2–43.2	46.1–63.2	53.9–72.1	47.6–74.9	46.1–55.5	
	NPN n/n (%)	15/37 (40.5)	40/117 (34.2)	69/126 (54.8)	66/104 (63.5)	28/45 (62.2)	218/429 (50.8)	< 0.001
	95% CI^b^	26.3–56.5	26.2–43.2	46.1–63.2	53.9–72.1	47.6–74.9	46.1–55.5	
	OR pre-notified vs. not (95% CI)	1.56 (0.60–4.10)	2.84 (1.70–4.74)	0.67 (0.42–1.06)	1.09 (0.56–2.14)	0.67 (0.23–1.99)	1.11 (0.85–1.45)	0.49^c^

^a^ Cochran-Armitage test for linear trend across years.

^b^ Wilson score interval.

^c^Fisher exact test, pooled. Mantel-Haenszel odds ratio adjusted for year: 1.22.

PN, pre notified; NPN, not-pre notified.

When comparing to reperfusion, pooled thrombolysis rates were 53.4% among pre-notified patients and 50.8% among those arriving without pre-notification, a difference that is not statistically significant (OR 1.11, 95% CI 0.85–1.45; Mantel-Haenszel adjusted for year, 1.22). Thrombolysis rates among pre-notified patients remained flat throughout (*p* = 0.96), while rates among non-pre-notified patients rose steeply, from 40.5% in 2022 to 62.2% in 2026 (*p* < 0.001).

### Functional dependency

Throughout the years of the study, the functional dependency was assessed using the mRS. This measure was taken at admission and discharge and the results showed that baseline functional status was high before arriving at the stroke center. In general, more than 80% of patients were functionally independent before the stroke event (mRS 0–2). Accordingly, a median of 0 in every year was found and no significant variation (*p* = 0.45) was evidenced.

At discharge, 448 of 814 evaluable patients (55.0%, 95% CI 51.6–58.4) were functionally independent. The median showed a mRS of 2 [IQR 1–4] in every year, again with no significant variation (*p* = 0.23) (Table 7a).

**Table 7a:** Modified Rankin Scale (mRS) scores at hospital admission and discharge, stratified by year.

mRS (rankin modified scale)	mRS value	2022 (N: 68)	2023 (N:253)	2024 (N:294)	2025 (N:162)	2026 (Q1) (N: 64)	Conglomerate (N: 841)	*p* ^a^
Hospital admission *n* (%)	mRS 0	39	172	210	110	49	580 (69.0)	
	mRS 1	8	42	37	21	5	113 (13.4)	
	mRS 2	5	11	18	12	3	49 (5.8)	
	mRS 3	6	19	16	14	6	61 (7.2)	
	mRS 4	6	4	11	3	1	25 (3.0)	
	mRS 5	0	3	1	2	0	6 (0.7)	
	Not specified	4	2	1	0	0	7 (0.8)	
	Median [IQR]	0 [0–2]	0 [0–1]	0 [0–1]	0 [0–1]	0 [0–0]	0 [0–1]	
	mRS 0–2, n/n^b^ (%)	52/64 (81.2)	225/251 (89.6)	265/293 (90.4)	143/162 (88.3)	57/64 (89.1)	742/834 (89.0)	0.45
	95% CI^c^	70.0–88.9	85.3–92.8	86.5–93.3	82.4–92.4	79.1–94.6	86.7–90.9	
Hospital discharge *n* (%)	mRS 0	14	52	51	29	14	160 (19.2)	
	mRS 1	9	71	53	25	7	165 (19.6)	
	mRS 2	3	26	52	32	10	123 (14.6)	
	mRS 3	7	28	51	31	11	128 (15.2)	
	mRS 4	8	30	37	17	8	100 (11.9)	
	mRS 5	2	22	15	14	2	55 (6.5)	
	mRS 6 (deceased)	5 (7.3)	24 (9.5)	32 (10.9)	12 (7.4)	10 (15.6)	83 (9.9)	
	Not specified	20	0	3	2	2	27 (3.2)	
	Median [IQR]	2 [0–4]	2 [1–4]	2 [1–4]	2 [1–4]	2 [1–4]	2 [1–4]	
	mRS 0–2, n/n^b^ (%)	26/48 (54.2)	149/253 (58.9)	156/291 (53.6)	86/160 (53.8)	31/62 (50.0)	448/814 (55.0)	0.23
	95% CI^c^	40.3–67.4	52.7–64.8	47.9–59.3	46.0–61.3	37.9–62.1	51.6–58.4	

^a^Cochran-Armitage test for linear trend across years.

^b^Denominator excludes patients with unspecified mRS. Missing data differed markedly by year (see Analysis).

^c^Wilson score interval.

When comparing both categories, shift from baseline was a substantive clinical finding. Independence fell from 89.0% before the event to 55.0% at discharge, meaning that approximately one in three previously independent patients left the hospital with a new dependency. This new dependency even though is remarkable, when analyzing the whole picture, it did not vary significantly across the study period (*p* = 0.23) and was maintained around 50%.

In the other hand, it should be emphasized that the Not specified category corresponds to patients in whom no mRS was documented in either the center's records or those of the referring hospital. This is independent of the NIHSS Not available category reported in Table 3, as documentation on the two scales was discretionary and unlinked: either score could be recorded in the absence of the other. Because the omission reflected the absence of a standardized documentation field rather than any characteristic of the patients themselves, the missingness is considered unrelated to outcome and that's why the mRS 0–2 row was calculated excluding these numbers and restricting the denominator only to evaluable patients. Considering the above, a sensitivity analysis shown in Table 7b was performed.

**Table 7b:** Sensitivity analysis of functional independence at discharge (mRS 0–2) to the handling of unspecified (NS) cases.

Scenario	2022 (N: 68)	2023 (N:253)	2024 (N:294)	2025 (N:162)	2026 (Q1) (N: 64)	Conglomerate (N: 841) n (%)	*p* ^a^
Complete-case (NS excluded—primary) % [95% Wilson confidence interval]	26/48 54.2% [40.3–67.4]	149/253 58.9% [52.7–64.8]	156/291 53.6% [47.9–59.3]	86/160 53.8% [46.0–61.3]	31/62 50.0% [37.9–62.1]	448/814 55.0% [51.6–58.4]	0.234
Scenario A: all NS = not independent (worst case)	26/68 38.2% [27.6–50.1]	149/253 58.9% [52.7–64.8]	156/294 53.1% [47.4–58.7]	86/162 53.1% [45.4–60.6]	31/64 48.4% [36.6–60.4]	448/841 53.3% [49.9–56.6]	0.986
Scenario B: all NS = independent (best case)	46/68 67.6% [55.8–77.6]	149/253 58.9% [52.7–64.8]	159/294 54.1% [48.4–59.7]	88/162 54.3% [46.6–61.8]	33/64 51.6% [39.6–63.4]	475/84 56.5% [53.1–59.8]	0.040
NS cases, *n* (%)	20 (29.4)	0 (0)	3 (1.0)	2 (1.2)	2 (3.1)	27 (3.2)	

^a^Cochran-Armitage test for linear trend across study periods.

Each cell shows the number of patients with mRS 0–2/evaluable denominator — proportion (%) [95% Wilson confidence interval]. The three scenarios differ only in how unspecified cases are treated: excluded from the denominator (complete-case), counted as not independent (Scenario A), or counted as independent (Scenario B). The bottom row shows the number and percentage of unspecified cases per period, calculated over the full annual cohort.

It should also be mentioned that mRS was not followed up at 90 days due to factors listed in the discussion.

### Mortality

Multiple variations in in-hospital mortality were observed but in general, the rate was just below 10 % (9.9% 95% CI 8.0–12.1) across the study period. Year by year it ranged from 7.4% to 15.6% without significant variation (*p* = 0.36) or linear trend (*p* = 0.39). The first quarter of 2026 year has the higher grade but derives from 10 deaths in a partial quarter of 64 patients, and its confidence interval (8.7–26.4) overlaps every other year (Table 8a).

**Table 8a:** In-hospital mortality cases, stratified by year.

Variable	Yes/No	2022 (N: 68)	2023 (N:253)	2024 (N:294)	2025 (N:162)	2026 (Q1) (N: 64)	Conglomerate (N: 841)	*p* ^a^
In-hospital death	Yes *n* (%)	5 (7.3)	24 (9.5)	32 (10.8)	12 (7.4)	10 (15.6)	83 (9.9)	0.39
	95% CI^b^	3.2–16.1	6.5–13.7	7.8–15.0	4.3–12.5	8.7–26.4	8.0–12.1	
	No *n* (%)	63	229	262	150	54	758 (90.1)	

^a^ Cochran-Armitage test for linear trend across years. ^b^ Wilson score interval.

The largest proportion of deaths corresponded to the male sex (53.0%) and to patients older than 65 years (68 out of 83 cases). Age was the strongest determinant of fatal outcome with a mortality of 11.8% (95% CI 9.4–14.7) compared with 5.3% (95% CI 3.0–9.2) in the 50–65 group, yielding an odds ratio of 2.24 (95% CI 1.26–4.01) and a significant trend across age strata. Surprisingly, the intermediate group of age, showed a correspondingly protective association (OR 0.43, 95% CI 0.22–0.83). Regarding sex, there was not a clear statistical relevant association between it and mortality.

Other relevant result was that mortality was lower among patients who received thrombolysis: 7.8% (95% CI 5.6–10.7) vs. 12.2% (95% CI 9.3–15.7) in untreated patients (OR 0.61, 95% CI 0.38–0.96). This find will be considered in fore coming paragraphs across the discussion.

The comorbidity most frequently associated with in-hospital death was HTN, which was present in more than 80% of fatal cases. This disease also increased risk of mortality (11.2% vs. 6.3% in normotensive patients; OR 1.91, 95% CI 1.05–3.47). In contrast, diabetes mellitus, dyslipidemia, and the combined metabolic profile showed no significant association (Table 8b).

**Table 8b:** Characteristics of fatal cases.

Variable	Category of variable	Deceased (N: 83)	Mortality in exposed, % (95% CI)^b, c^	OR (95% CI)^d^	*p* ^e^
Sex (*n* %)	Female	39 (47.0)	9.2 (6.8–12.3)	0.86 (0.55–1.35)	0.56
	Male	44 (53.0)	10.6 (8.0–13.9)	1.00 (ref)	
Age (*n* %)	< 50 years	4 (4.8)	7.0 (2.8–16.7)	0.67 (0.24–1.91)	0.64
	50–65 years	11 (13.2)	5.3 (3.0–9.2)	0.43 (0.22–0.83)	0.011
	>65 years	68 (81.9)	11.8 (9.4–14.7)	2.24 (1.26–4.01)	0.004
Thrombolysis (*n* %)	Yes	34 (41.0)	11.8 (9.4–14.7)	0.61 (0.38–0.96)	0.037
	No	49 (59.0)	12.2 (9.3–15.7)	1.00 (ref)	
Medical history (*n* %)	Hypertension	69 (83.1)	11.2 (8.9–14.0)	1.91 (1.05–3.47)	0.036
	Diabetes mellitus	25 (30.1)	11.6 (8.0–16.6)	1.29 (0.78–2.12)	0.35
	Dyslipidemia	26 (31.3)	9.8 (6.8–14.0)	0.99 (0.61–1.61)	1.00
	HTN + DM + dyslipidemia	8 (9.6)	7.1 (3.6–13.4)	0.66 (0.31–1.41)	0.40

HTN, arterial hypertension; DM, diabetes mellitus.

χ^2^ of independence = 4.35, *df* = 4, *p* = 0.36.

^b^Wilson score interval.

^c^Proportion of patients with the characteristic who died; denominators taken from Tables 2, 4. Estimates are unadjusted; see limitations.

^d^Odds of death among patients with the characteristic vs. those without, pooled across years.

^e^Fisher exact test.

## Discussion

This study describes an organized stroke care network implemented in a small department of Colombia and its follow-up over 51 months. Throughout the study period, two major outcomes were assessed: in-hospital mortality and functional dependence. About fatality during internation, this represented 9.9% of the population (95% CI: 8.0–12.1) and functional independence at discharge (mRS 0–2) was 55.0% (95% CI: 51.6–58.4). Neither of these measures changed significantly over the years, and this stability occurred alongside a substantial increase in reperfusion rates and a marked fluctuation in patient volume like will be discussed later.

The statistical concordance between the prevalence of modifiable risk factors observed in the patients treated at the center and those reported in other studies (O'Donnell et al., 2016; Boehme et al., 2017) supports the premise that cerebral infarction may be preventable. In the present cohort, HTN emerged as the most prevalent risk factor (73.1%), followed by dyslipidemia (31.5%) and DM (25.6%). Notably, the combined presence of hypertension, type 2 diabetes, and dyslipidemia was more common than smoking (13.4% vs. 2.6%). While this finding could suggest a high frequency of metabolic syndrome in the Coffee Triangle region vs. smoking, it most likely reflects underreporting of the habit in clinical records. The similar could explain why hypothyroidism rose from 0% in 2022 to 11.9% in 2024. Urban origin followed the same pattern, rising abruptly from 50.0% to above 93% in a manner compatible with either a shift in referral catchment following certification or a change in recording practice.

Annual patient volume also varied for identifiable operational reasons rather than epidemiological ones. The increase following certification reflected the network becoming the regional reference for acute stroke, while the decline in 2025 followed three concurrent factors: the entry into operation of additional stroke-treating centers in the region, preventive maintenance and unscheduled equipment failures that required temporary diversion of patients, and the termination of a strategic agreement with insurers that had channeled referrals to this center. Despite that, admission NIHSS remained stable throughout the study period (*p* = 0.58), indicating that these changes affected how many patients were treated but not the severity of those who arrived. Given that admission severity showed no significant variation across time, the improvement observed at discharge is difficult to attribute to a shift toward milder cases reaching the institution and it is more consistent with an improvement in the care pathway (mean 2 over the years, *p* = 0.23).

Other relevant finds were regarding fluctuations in door-to-needle time that are explained by both logistical and clinical features. For instance, the rise in patient volume in 2023 and 2024, along with the diversification and case complexity in 2025, added to the simultaneous presentation of multiple patients and the unavailability of technical resources and specialized personnel all the time, could account for some of the increases in thrombolysis times. Regardless, the 60-min target was never exceeded across any of the years analyzed.

As door-to-needle times increased, so did the rate of fibrinolysis year over year. Overall, the sustained inclination toward eligibility for pharmacological revascularization therapy reflects not only earlier patient access to specialized medical care, but also more timely stroke detection, which may itself translate into higher pre-notification rates. Nevertheless, when pre-notified patients are compared with those who ultimately underwent thrombolysis, an inverse relationship has emerged over time. It was found that pre-notification declined significantly from 57.1% in 2024 to 29.7% in early 2026 (*p* < 0.001), while thrombolysis among pre-notified patients remained stable (*p* = 0.96) and rates among unannounced arrivals rose from 40.5% to 62.2% (*p* < 0.001). The advantage conferred by pre-notification, clearly present in 2023 (OR 2.83, 95% CI 1.70–4.74), was no longer detectable thereafter. A plausible explanation for this is that more patients reached the center directly as regional awareness of stroke as a time-dependent emergency grew, while in-hospital processes improved enough to treat them at comparable rates.

When comparing against national records, it was found that in the Colombian RES-Q analysis (Bayona et al., 2025) of 7,963 patients an intravenous thrombolysis rate of 14.5% was reported and a median door-to-needle time of 53 min, with 33.4% achieving mRS 0–2 at discharge. The thrombolysis rate of 52.1% of the study (95% CI 48.7–55.4) and independence rate of 55.0% exceed these substantially, though the comparison is not totally even since the national registry aggregates institutions of heterogeneous complexity, including centers without thrombolysis capability, while this cohort comes from a single certified stroke center. Similar patterns have been described elsewhere in the region. A Peruvian RES-Q cohort (Gallo-Guerrero et al., 2026) found functional independence at discharge in 50.7% of thrombolyzed patients vs. 38.3% of untreated patients, with thrombolysis remaining an independent predictor of favorable outcome after adjustment. Our unadjusted finding of lower mortality among thrombolyzed patients (7.8% vs. 12.2%; OR 0.61, 95% CI 0.38–0.96) points in the same direction but is subject to confounding by indication and cannot be read as a treatment effect. In Mexico, the ResISSSTE Cerebro hub-and-spoke model achieved a 35% thrombolysis rate with a median door-to-needle time of 76 min; our door-to-needle times remained below 40 min throughout (Bonifacio-Delgadillo et al., 2023), though recorded as means without dispersion measures and therefore not formally comparable.

Safety outcomes support the expansion of reperfusion as does the mortality associated with it. The results showed that thrombolyzed patients had a OR of 0.61 (0.38–0.96) and a *p* statically significant (0.037) regarding fatality. About complications after stroke and reperfusion therapies, hemorrhagic transformation occurred in 4.5% (95% CI 3.3–6.1) of the cohort and in 7.3% of thrombolyzed patients. Symptomatic hemorrhage was documented in only 4 of 38 transformations −0.48% of the cohort (95% CI 0.19–1.22).

Complication rates did not vary significantly across years despite the use of a mean thrombolytic dose in the center slightly above the Colombian national average and compares favorably with the margins reported in large international cohorts such as the SITS-MOST registry (Wahlgren et al., 2007). It is worth mentioning that the thrombolysis comparison is unadjusted for admission severity and therefore subject to confounding by indication, as more severe strokes are both more likely to be treated and more likely to bleed. Additionally, sedation precluded NIHSS assessment in 10 patients with hemorrhagic transformation, so the count of symptomatic events represents a minimum rather than a definitive figure. Malignant edema was rare and was presented in less than 5% of the total population (3.9).

Finally, it should be said that the changes in the mRS at discharge and mortality were given within the framework of a model that has been sustainable over the years and uses resources that were already available. The CRUE maintains a radio operator on duty 24 h, so the referral code that follows each case discussion is issued without additional staffing. The physicians participating in the network work rotating shifts, which distributes the burden of responding across a wider group rather than concentrating it on a few individuals, and the messaging application through which cases are discussed is almost universally available and carries negligible cost. Because physicians in their mandatory social service year rotate frequently through referring institutions, periodic training has been essential to maintain protocol adherence despite continuous personnel turnover; this training requirement is the recurring cost of the model, and it is modest compared with the infrastructure that a formal telestroke system would demand. Scalability would benefit from a dedicated application or platform, which could automate case registration and referral coding and thereby reduce the human resource currently required at each step, though such a development would also resolve the data protection questions that consumer messaging leaves open. Despite the barriers and gaps in access to reperfusion therapies for stroke in low- and middle-income countries (LMICs)—as well as in access to quality care—as noted in the studies by Sikakulya et al. (2026) and Rahbar et al. (2022).

The relevance of this experience lies in the evidence that organized regional networks for integrated stroke management are achievable in middle-income settings without capital investment, and their development in other represents a realistic path toward reducing the disability burden of cerebrovascular disease in Colombia and other low- and middle-income countries. The replicability of the findings shown will require prospective multicenter evaluation.

## Conclusions

The present study shows that the strengthening and establishment of essential stroke care centers lead to earlier and more accurate detection of acute cerebral infarction, and consequently to faster access to specialized medical care and reperfusion therapies. These changes in the treatment pathway could be translated in some cases to lower mortality and greater functional independence.

## Limitations and strengths

The Red ACV-Quindío strategy was not initially prepared for the flow of patients it would have. Since the WhatsApp^®^ group was only conformed by a neurologist and a few doctors in rural areas, the increase in patient volume and complexity lead to the addition of the CRUE's radio operators and the emergency departments of all the region. This change in the conformation of the group allowed the expansion of the project not compromising the speed at which the patients were directed or its safety. In the future, an assessment of the integrity of the network should be done.

Although medical coverage (insurer) limited access to the center and decreased the number of patients in a particular year, as a rule, all those with a symptom evolution time of less than 24 h were accepted regardless of whether they were candidates for pharmacological thrombolysis. The distinction was made, as previously mentioned, due to the suggestion of international guidelines to maintain strict neurocritical care during the acute period of the disease. The omission of thrombectomy data is due not to its unavailability but to its being reserved for future research.

Even though monitoring functional dependency would have been a very noteworthy aspect of the Red ACV-Quindío strategy, the mRS measurement at 90 days was not done because it requires several resources: time, personnel and access to contact data that are not readily available or change constantly.

Although the data presented has methodological and inherent limitations, comparisons with national or regional records were not made due to their absence, which constitutes one of the research's greatest strengths.

## Data Availability

The raw data supporting the conclusions of this article will be made available by the authors, without undue reservation.
